# Supplementary material for: Correlation of Mercury Occurrence with Age, Elemental Composition, and Life History in Sea-Run Food Fish from the Canadian Arctic Archipelago’s Lower Northwest Passage
Source: Foods. 2021 Oct 29;10(11):2621. doi: 10.3390/foods10112621 (PMC8618063; doi:10.3390/foods10112621)
Supplement: Supplementary file 1 [file foods-10-02621-s001.zip › foods-1403299-supplementary.pdf]

foods

MDPI

Supplemental Information:

Correlation of Mercury Occurrence with Age, Elemental Composition, and Life History in Sea-Run Food Fish from the Canadian Arctic Archipelago's Lower Northwest Passage

Iris Koch [1,2], Pranab Das [1], Bronte E. McPhedran [1], John M. Casselman [1], Kristy L. Moniz [1], Peter van Coeverden de Groot [1], James Qitsualik [3], Derek Muir [4], Stephan Schott [5] and Virginia K. Walker [1,6,*]

1 Department of Biology, Queen's University, Kingston, ON K7L 3N6, Canada; koch-i@rmc.ca (I.K.); pd50@queensu.ca (P.D.); bronte.mcphedran@queensu.ca (B.E.M.); john.casselman@queensu.ca (J.M.C.); monizk@queensu.ca (K.L.M.); degrootp@queensu.ca (P.v.C.d.G.)
2 Environmental Services Group, Royal Military College of Canada, Kingston, ON K7K 4B4, Canada
3 Gjoa Haven Hunters and Trappers Association, Gjoa Haven, NU X0B 1J0, Canada; gjoa@krwb.ca
4 Environment and Climate Change Canada, Canada Center for Inland Waters, Burlington, ON L7S 1A1, Canada; derek.muir@ec.gc.ca
5 School of Public Policy and Administration, Carleton University, Ottawa, ON K1S 5B6, Canada; StephanSchott@Cunet.Carleton.Ca
6 School of Environmental Studies, Queen's University, Kingston, ON K7L 3N6, Canada
* Correspondence: walkervk@queensu.ca Tel.: +1-613-533-6123

Otolith analysis- supplemental methods

Two independent age interpretations were conducted on each otolith section at different times and on each occasion a confidence rank was assigned (9, extreme confidence; 1, no or little to no confidence). If the age interpretations disagreed, another independent age interpretation was conducted, and if necessary due to disagreement, the interpretation with the highest confidence rank was recorded. Age interpretations provided a calcified structure age (CSA), which included enumeration of the annuli plus an edge code following the calcified structure age interpretation system (CSAIS), with this technique describing the edge of the otolith in relation to annulus formation (Casselman, 1987, 2015). Taking into consideration the time of annulus formation, CSAIS allows the conversion to year-class, or calendar age with two successive annuli delineating one year of otolith growth (Casselman, 1987). In some slow-growing fish, it was challenging to resolve the zonation near the edge of the otolith sections, necessitating additional analysis, particularly for older lake whitefish and lake trout. In such cases, the surface of the otolith was etched with 2% hydrochloric acid and an acetate acetone-softened impression of the etched surface was made as described (Casselman and Gunn, 1992), with zonation on the acetate replicate is usually much better defined (Supplemental Fig. 1).

In order to explore the incremental growth (fork length/age) of each of the four salmonid species, while correcting for age, mean fork length/age values for each age were log standardized to produce a standard growth-at-age curve representative of the entire data set (see Supplemental Table 1).

References for Supplemental Material:

Casselman, J. M. Determination of age and growth. In *The biology of fish growth*; Weatherly, A. H.; Gill, H. S., Eds.; Academic Press: London, **1987**, 209-242.

Casselman, J.M. A techniques manual outlining general procedures for preparing thin sections of fish otoliths for age and growth interpretation. Special publication of *AFishESci Inc.*, Mallorytown, Ontario, **2015**, 30 p.

Casselman, J.M. and Gunn, J.M. Dynamics in year-class strength, growth, and calcified structure size of native lake trout (*Salvelinus namaycush*) exposed to moderate acidification and whole-lake neutralization. *Can. J. Fish. Aquat. Sci.* **1992**, 49 (Suppl. 1), 102-113.

Supplemental Table S1. Incremental fork length (FL) growth standards for the salmonids sampled, developed from the relationship mean FL·Age^{-1} for 197 Arctic char, 136 lake trout, 100 lake whitefish, and 98 cisco, with the linear growth standard in mm·yr^{-1}.

Age (yr)	Arctic char	Lake trout	Lake whitefish	Cisco	Age (yr)	Lake trout	Lake whitefish
2				77.7	33	21.2	13.3
3				60.8	34	20.8	13.0
4			74.9	51.1	35	20.4	12.7
5	96.7		62.4	44.6	36	20.0	12.4
6	84.8		53.7	40.0	37	19.7	12.1
7	75.8		47.4	36.4	38	19.4	11.9
8	68.9	53.0	42.5	33.6	39	19.0	11.6
9	63.2	49.1	38.6	31.3	40	18.7	11.4
10	58.6	45.9	35.4	29.3	41	18.4	11.1
11	54.7	43.1	32.7	27.7	42	18.1	10.9
12	51.4	40.8	30.5	26.3	43	17.9	10.7
13	48.5	38.7	28.5	25.0	44	17.6	10.5
14	46.0	36.9	26.9	23.9	45	17.4	10.3
15	43.7	35.3	25.4	22.9	46	17.1	10.1
16	41.7	33.9	24.1	22.1	47	16.9	10.0
17	39.9	32.6	22.9	21.3	48	16.6	
18	38.3	31.4	21.9	20.5	49	16.4	
19	36.9	30.3	20.9	19.9	50	16.2	
20	35.5	29.2	20.1	19.3	51	16.0	
21	34.3	28.4	19.3	18.7	52	15.8	
22	33.1	27.6	18.5	18.2	53	15.6	
23	32.1	26.8	17.9	17.7	54	15.4	
24	31.1	26.1	17.3	17.3	55	15.2	
25	30.2	25.4	16.7	16.8	56	15.1	
26	29.4	24.7	16.2	16.4	57	14.9	
27	28.6	24.1	15.7	16.1	58	14.7	
28	27.8	23.6	15.2	15.7	59	14.6	
29	27.2	23.1	14.8	15.4	60	14.4	

30	22.6	14.4	15.1	61	14.2
31	22.1	14.0		62	14.1
32	21.6	13.6			

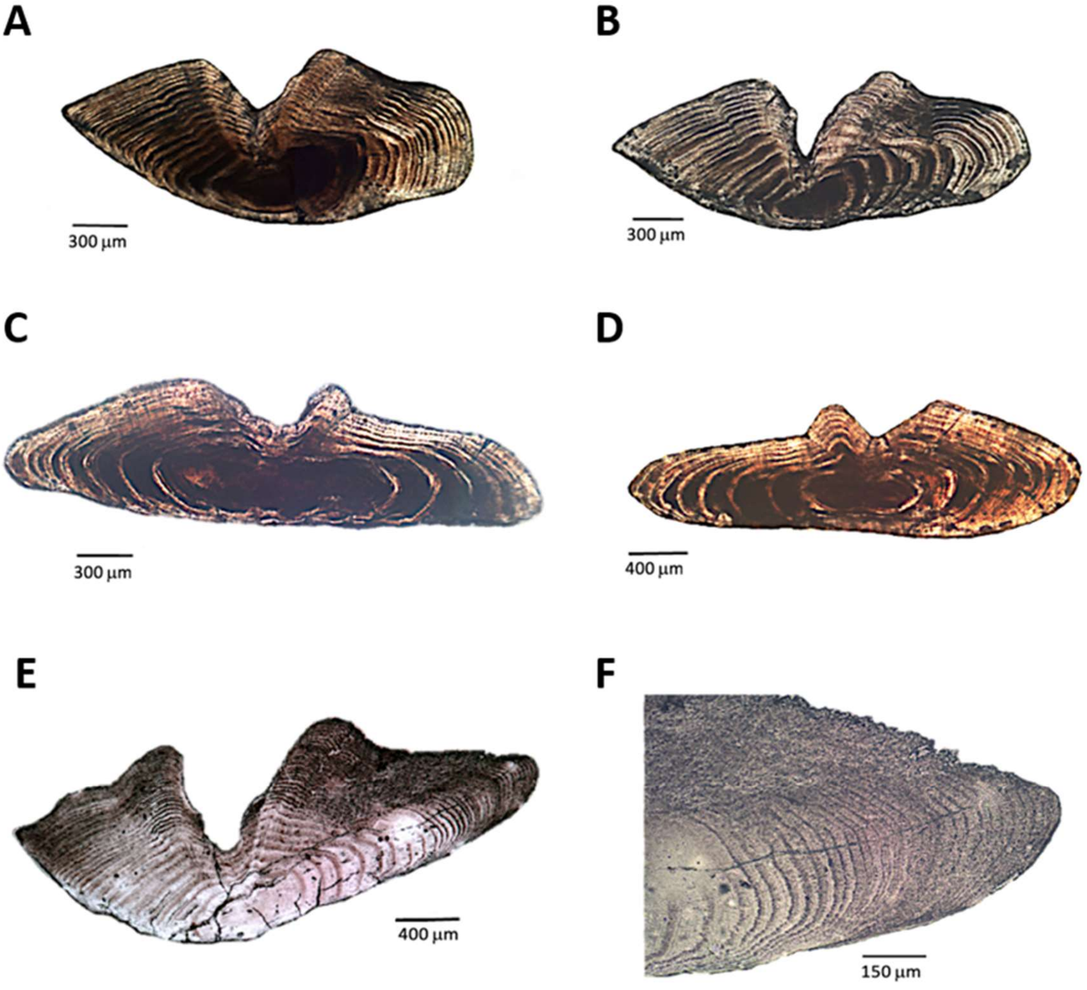

Supplemental Figure S1. Otolith sections of long-lived, slow-growing Arctic fish typical of images used to assess otolith age. **A**) Arctic char otolith section assessed age 17 years, calcified structure age (CSA) = 17++, caught December 8, 2017, from the 2000 year-class, FL = 584 mm, body weight 2,044 g. **B)** Arctic char otolith section assessed age 14 years, CSA = 14++, caught December 8, 2017, from the 2008 year-class, FL = 532 mm, body weight 1,614 g. **C**) Lake whitefish otolith section assessed age 9 years, CSA = 8++, caught May 18, 2016, from the 2007 year-class, FL = 319 mm, body weight 380 g. **D**) Cisco spp. otolith section assessed age 8 years, CSA = 8++, caught December 18, 2016, from the 2008 year-class, FL = 360 mm, body weight 536 g. **E**) Acetate replica of a lake trout otolith section assessed age 40 years, CSA = 40++, caught December 18, 2016, from the 1976 year-class, FL = 700 mm, body weight 4,450 g.
F) Ventral tip of acetate replica of the otolith of the lake trout illustrated in E) showing the precise and closely spaced zonation of annual increments deposited in the otolith from 1984 to the present. The image illustrates the fine detail available in the acetate replicas (see Supplemental material), necessary to acquire accurate age and growth interpretation of the otoliths of old, slow-growing fishes.

Elemental analysis - detection/reporting limits

Detection limits, provided as reporting limits for analysis conducted by the CALA accredited laboratories, QASU and Flett Research Ltd, are listed in Supplemental Table 2. Limits of detection were established using 7 (MeHg) or 9 (THg, elements) method blanks, and reporting limits are a practical multiplier on the limits of detection (typically 3).

Supplemental Table S2. Detection/reporting limits for elements analyzed in the sampled fish tissue

Element	Detection/reporting limit (mg/kg dry weight)
Total mercury	0.005
Methyl mercury	0.016
Aluminium	<10
Antimony	<0.1
Arsenic	<0.5
Barium	<0.1
Beryllium	<0.005
Boron	<10
Cadmium	<0.005
Calcium	<20
Cerium	<0.020
Chromium	<0.1
Cobalt	<0.01
Copper	<0.5
Dysprosium	<0.0025
Erbium	<0.0025
Europium	<0.0025
Gadolinium	<0.0025
Gallium	<0.05
Germanium	<0.5
Holmium	<0.0025
Iridium	<0.01
Iron	<10
Lanthanum	<0.005
Lead	<0.05
Lithium	<0.5
Lutetium	<0.0025
Magnesium	<2.0
Manganese	<0.2

Molybdenum	<0.05
Neodymium	<0.010
Nickel	<0.05
Niobium	<0.005
Phosphorus	<20
Platinum	<0.005
Potassium	<15
Praseodymium	<0.0025
Rhenium	<0.005
Rhodium	<0.005
Rubidium	<0.05
Ruthenium	<0.005
Samarium	<0.0025
Selenium	<0.1
Silver	<0.010
Sodium	<15
Strontium	<0.2
Sulfur	<25
Tantalum	<0.0025
Terbium	<0.0025
Tellurium	<0.01
Thallium	<0.0025
Thulium	<0.0025
Tin	<0.1
Titanium	<0.2
Tungsten	<0.01
Uranium	<0.0025
Vanadium	<0.02
Ytterbium	<0.0025
Yttrium	<0.025
Zinc	<2.0
Zirconium	<0.05

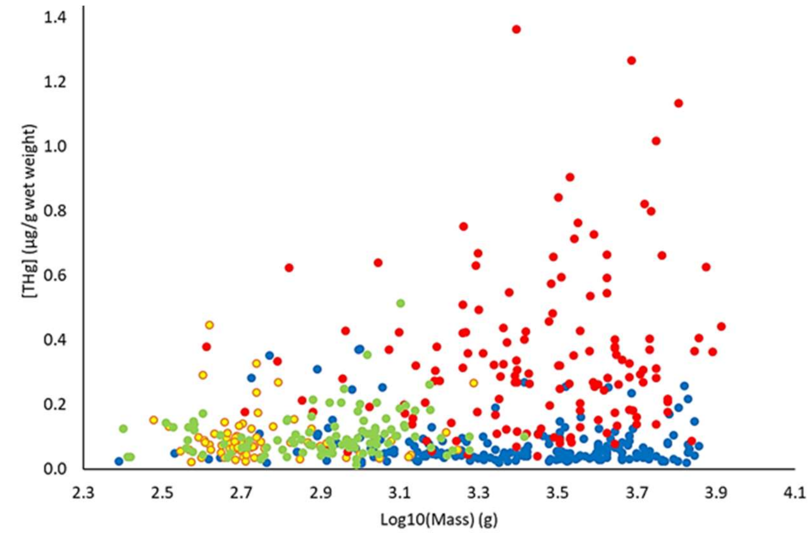

Supplemental Figure S2. A scatter plot showing total mercury (THg) in Arctic char (blue), lake trout (red), lake whitefish (light green) and cisco (yellow) in relation to mass ($\log_{10}$ grams) in fish (individuals represented as a single point on the graph) caught in this region of Nunavut, Canada. As shown in the main text Table 2, only lake trout and lake whitefish show a significant relationship with this factor.

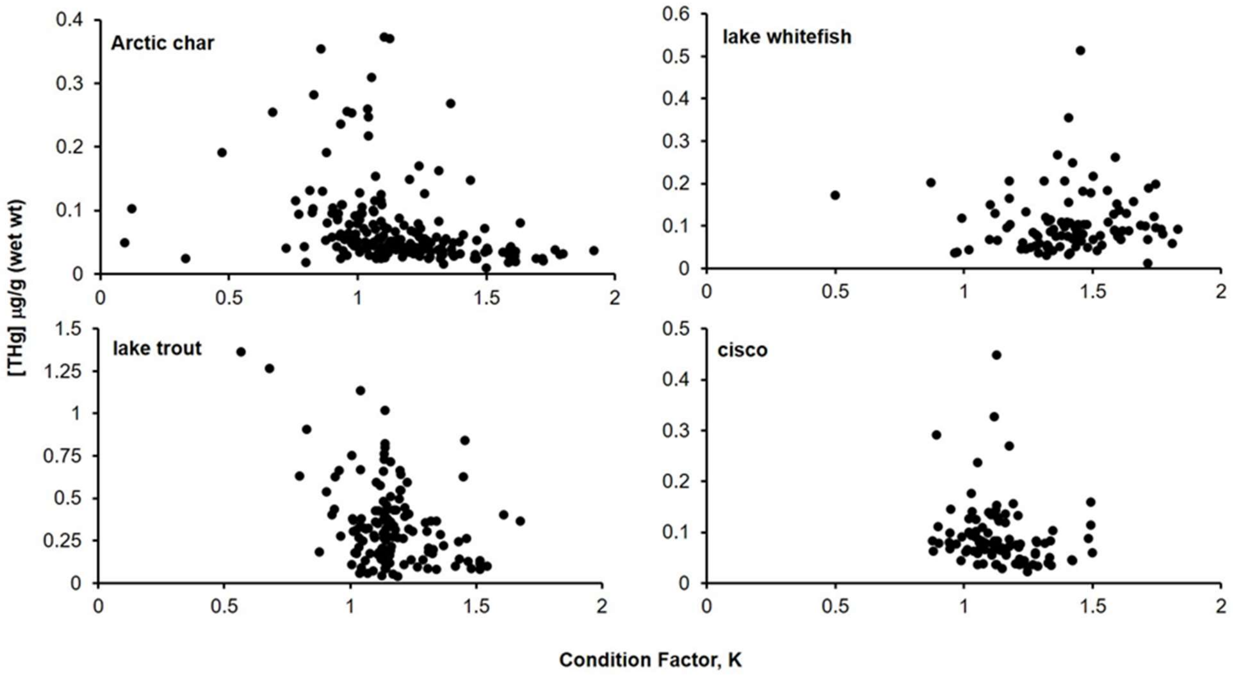

Supplemental Figure S3. Scatter plots showing total mercury (THg) with respect to condition or K ($K = W/L^3$; see Methods) in the four salmonid types, with individual fish shown as dots. Note that the Y-axes representing THg levels change for each salmonid but the axis for lake trout is approximately one-third the scale as shown for the other fish. As indicated (text Table 2), only Arctic char and lake trout show a significant relationship with this factor.

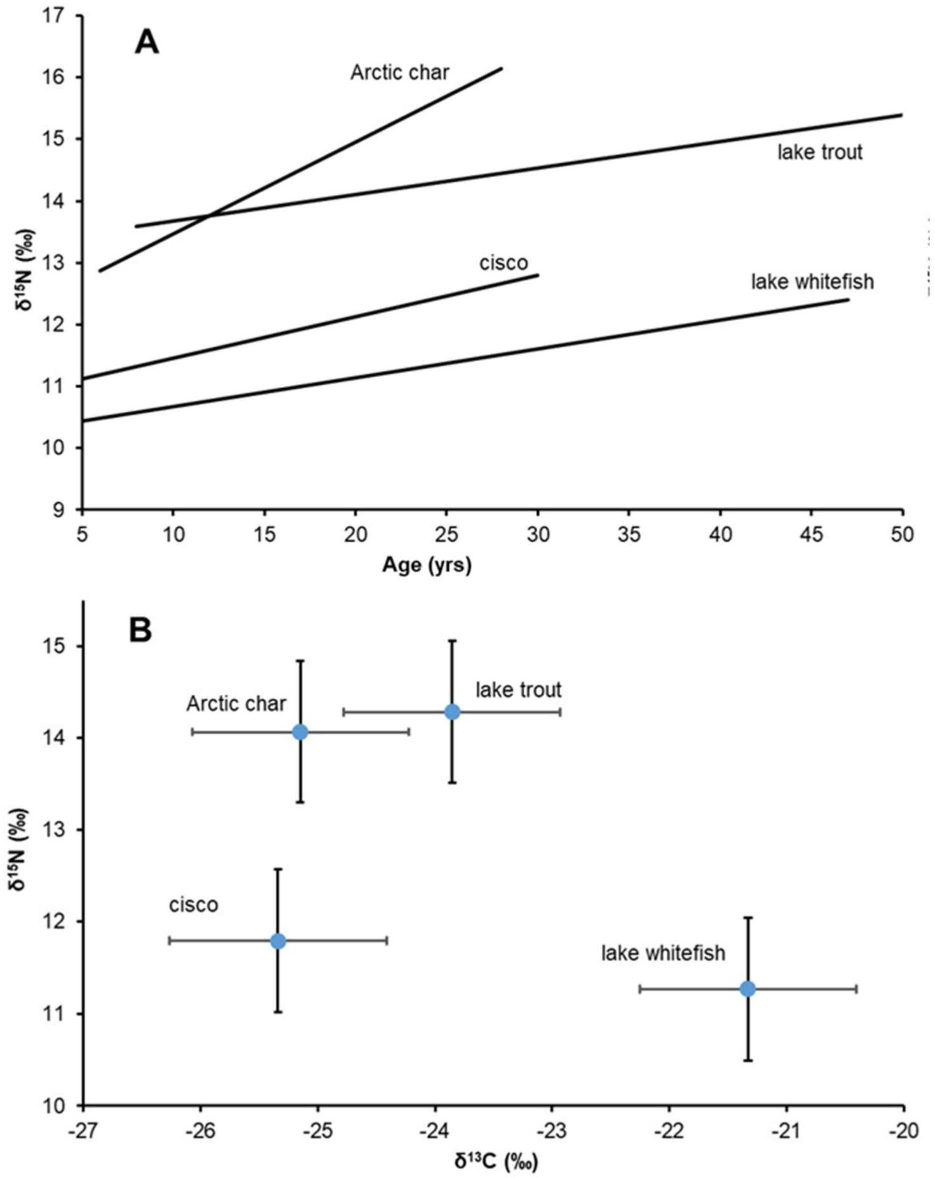

Supplemental Figure S4. Nitrogen and carbon isotope ratio signature relationships in salmonids from fishing sites located on or near King William Island in Nunavut (A) Linear regressions of δ^{15}N (‰) and age for Arctic char, lake trout, cisco, and lake whitefish, with significant differences in the calculated regressions for δ^{15}N and age in Arctic char ($F(1,101)=23.93$, $r^2 = 0.19$, $p<0.01$), lake trout ($F(1,78)=9.37$, $r^2=0.11$, $p<0.01$), cisco ($F(1,64)=21.30$, $r^2=0.25$, $p<0.01$) and lake whitefish ($F(1,54)=12.48$, $r^2=0.19$, $p<0.01$). (B) Mean δ^{13}C and δ^{15}N signatures ± 1 standard deviation for Arctic char (n= 102), cisco (n= 65), lake trout (n= 79), and lake whitefish (n=55). Changes in δ^{15}N (‰) were significantly and positively related to δ^{13}C values for lake trout ($F(1,78)=8.22$, $r^2=0.10$, $p<0.05$) and significantly and negatively related for cisco ($F(1,64)=7.37$, $r^2=0.008$).10, $p<0.05$).

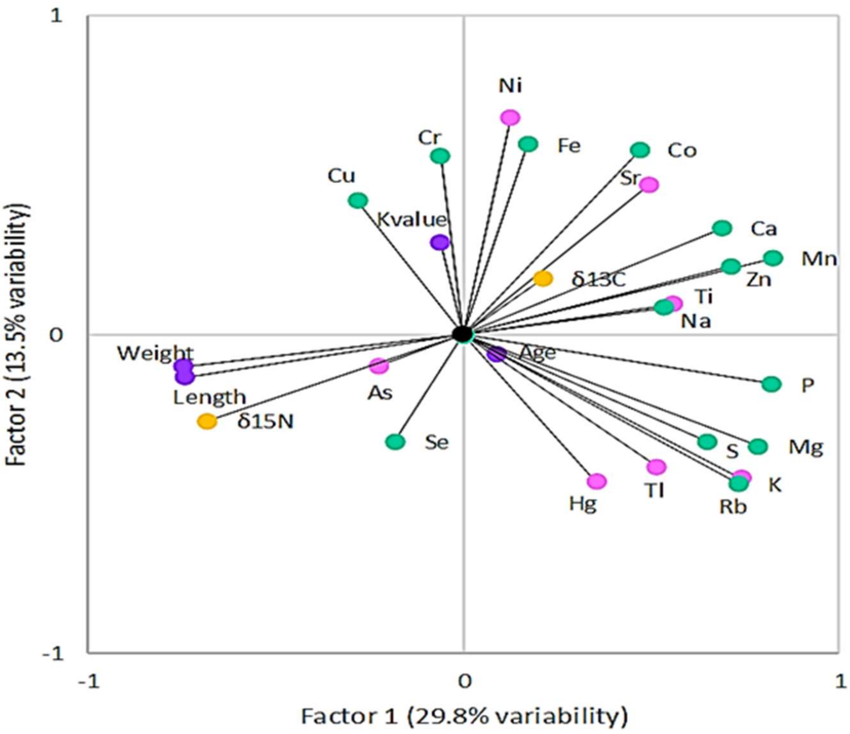

Supplemental Figure S5. Exploratory principal component analysis (PCA) factor loading plot for the variables used in the correlation analysis (Spearman, number of observations = 321) shown in text Fig. 2. All salmonid samples were grouped for the PCA analysis and included essential and non-essential elements (green and pink dots, respectively), carbon ($\delta^{13}C$) and nitrogen ($\delta^{15}N$) isotopes (yellow) and physical characteristics (purple) including weight, length, and condition (K value).

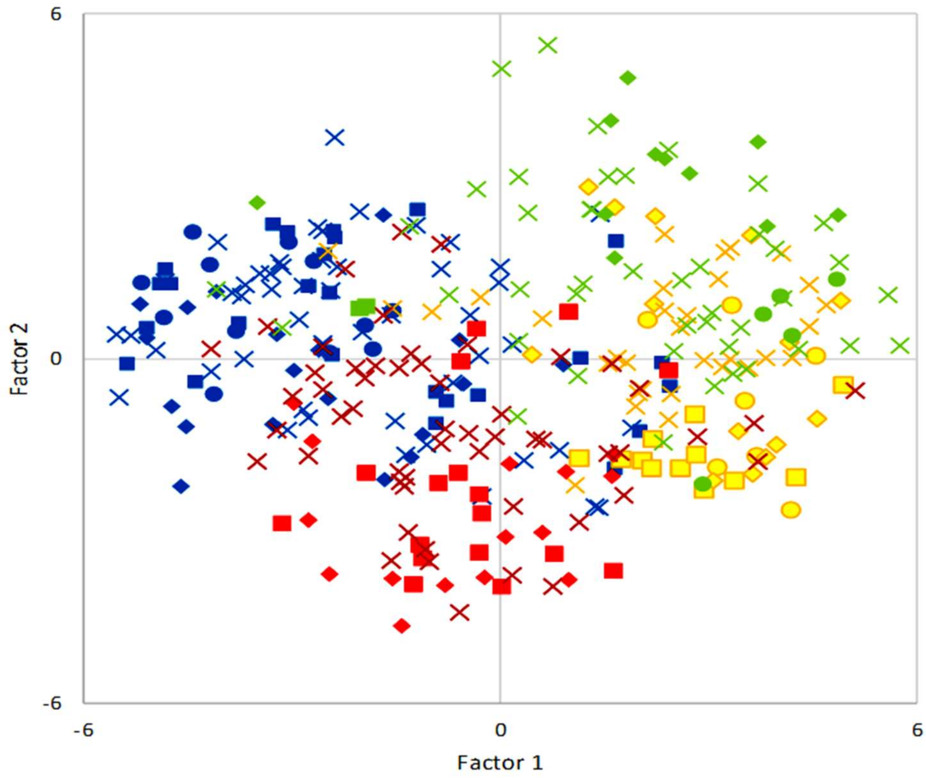

Supplemental Figure S6. Principal components analysis (PCA) showing the first two factors (factor 1 and 2 at 29.8% and 13.5% , respectively, for a sum of 43% of the variance) with the different sampled salmonids plotted so that the segregation of the different species with respect to these factors can be more easily visualized. Different symbols represent the sampling location (shown in text Fig. 1) with geographic group 1, 2, 3 and 4 represented by dots, boxes, diamonds and Xs, respectively. Each salmonid type was assigned a different colour with Arctic char (blue symbols), lake whitefish (green symbols), cisco (yellow symbols) and lake trout (red symbols). Figure 3 in the main text is the same except that the symbols for the different fishing sites above, were not included so that differences between the salmonids, irrespective of sampling site, could be emphasized.

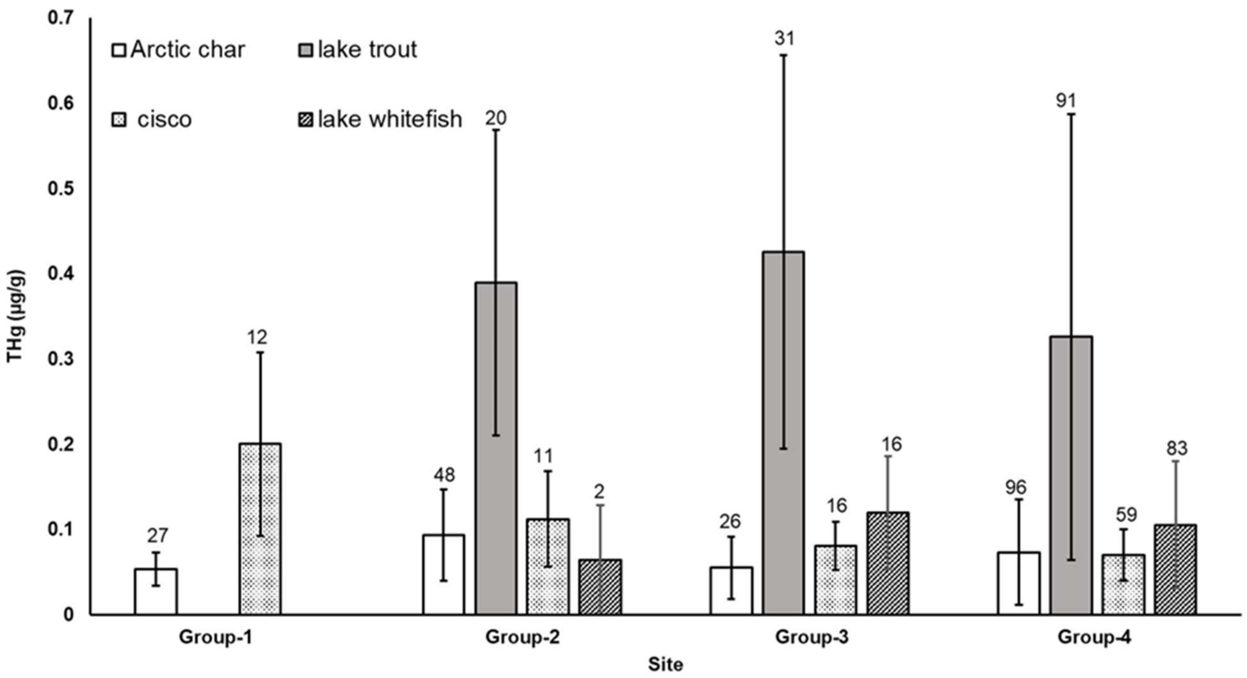

Supplemental Figure S7. Mean total mercury (THg) levels for the four salmonids caught at different fishing sites grouped according to their locations on or near *Qikiqtaq* (King William Island). Fishing sites are generally grouped from north to south along the X-axis (see text Fig. 1), with Group-1 (north King William Island; Port Parry), Group-2 (south and middle King William Island; Koka, Merilik, KWI Weir), Group-3 (east of the south end of King William Island; Murchison River) and Group-4 (south of King William Island: Backhouse Point, Back River, Legendary River, and the Lakes West of Chantry), with not all sites indicated. Inuktituk names are listed in Table 1. Mean THg levels were calculated using average ages of 0-20, 21-30, 31-40, 41-50 and 51-65 years for Arctic char, lake trout, cisco and lake whitefish at each fishing site grouping. Numbers of samples from each group are indicated above each of bars representing the standard deviation**.**
